# Associations of sustained smoking and smoking cessation with work-related outcomes: a longitudinal analysis

**DOI:** 10.1007/s00420-020-01598-3

**Published:** 2020-11-10

**Authors:** Sigrid A. Troelstra, Cécile R. L. Boot, Janneke Harting, Goedele A. Geuskens, Anton E. Kunst, Allard J. van der Beek

**Affiliations:** 1 Department of Public and Occupational Health, Amsterdam Public Health Research Institute, Amsterdam UMC, VU University, Van der Boechorstraat 7, 1081 BT Amsterdam, The Netherlands; 2 Department of Public Health, Amsterdam Public Health Research Institute, Amsterdam UMC, University of Amsterdam, Amsterdam, The Netherlands; 3 Netherlands Organisation for Applied Scientific Research TNO, Leiden, The Netherlands

**Keywords:** Smoking, Smoking cessation, Sickness absence, Work productivity, Work ability, Older workers

## Abstract

**Purpose:**

The aim of this study was to assess the association between sustained smoking and quitting with work-related outcomes among older workers.

**Methods:**

We categorized a sample of older employees into non-smokers, sustained smokers and quitters. Multivariable regression models were used to test longitudinal associations of sustained smoking and smoking cessation with sickness absence, productivity loss and work ability.

**Results:**

We included 3612 non-smokers, 673 sustained smokers and 246 quitters. Comparing sustained smokers to non-smokers, we found higher (but not statistically significant) sickness absence for sustained smokers [1.01, 95% confidence interval (CI) − 0.16–2.17]. We did not find differences in productivity loss (OR 0.82, 95% CI 0.60–1.13) and work ability (0.05, 95% CI −0.05–0.15). For employees with a relatively high physical health at baseline, comparing quitters to sustained smokers, we found higher (but not statistically significant) productivity loss for quitters (OR 2.23, 95% CI 0.94–5.31), and no difference in sickness absence (0.10, 95% CI − 2.67–2.87), and work ability (− 0.10, 95% CI −  0.36–0.16). For employees with a relatively low physical health at baseline, comparing quitters to sustained smokers, we found a statistically significant lower work ability (− 0.31, 95% CI − 0.57–0.05), and no difference in sickness absence (2.53, 95% CI − 1.29–6.34) and productivity loss (OR 1.26, 95% CI 0.66–2.39).

**Conclusions:**

We found no evidence that sustained smokers have less favorable work-related outcomes than non-smokers or that quitters have more favorable work-related outcomes than sustained smokers. The benefits of smoking cessation for employers might take a longer time to develop.

## Background

Smoking remains a major worldwide public health threat (World Health Organization 2018). Next to an increased risk of premature death, smokers experience more health problems compared to non-smokers. Smokers have an increased risk of developing cardiovascular diseases, chronic obstructive pulmonary disease (COPD), and various types of cancer (Kõks et al. 2018; Taghizadeh et al. 2016). Furthermore, smoking is associated with a lower quality of life (Coste et al. 2014; Goldenberg et al. 2014) and mental health problems such as depression and anxiety (Fluharty et al. 2016; Prochaska et al. 2017). The negative health impact of smoking becomes more prominent with older age (Nicita-Mauro et al. 2008; Østbye and Taylor 2004).

Through its negative influence on physical health, mental health and quality of life, smoking can influence employability of workers. Employability can be captured by different work-related outcomes, such as sickness absence, work productivity and work ability (Tarro et al. 2020). According to two systematic reviews, smoking is associated with an increase in sickness absence rates (Troelstra et al. 2020; Weng et al. 2013). Smoking is also associated with a decrease in work productivity (Berman et al. 2014; Bunn et al. 2006; Halpern et al. 2001; Sherman and Lynch 2013). Most studies found negative associations between smoking and work ability [i.e., self-assessed work ability in relation to an individual’s resources and job demands (Van den Berg et al. 2009)] (Airila et al. 2012; Augusto et al. 2015; Mohammadi et al. 2014; Tuomi et al. 1991), while one study did not find an association (Fischer and Martinez 2013). Furthermore, smoking is associated with a risk of early exit from work (Bengtsson and Nilsson 2018; Husemoen et al. 2004). Few studies have compared the association between smoking and different work-related outcomes (Berman et al. 2014; Bunn et al. 2006; Halpern et al. 2001; Sherman and Lynch 2013; Tsai et al. 2005), and to our knowledge, no study has included sickness absence, work productivity, and work ability.

The workplace is a setting that has the potential to reach large groups of people, to have higher intervention participation rates compared to non-occupational settings, and to encourage peer-support and positive peer-pressure (Cahill and Lancaster 2014). A Cochrane review on the effect of workplace smoking cessation interventions found strong evidence for the effectiveness of workplace based smoking cessation interventions (Cahill and Lancaster 2014). More knowledge about the relation between smoking cessation and work-related outcomes, especially among older workers, could support the further development and implementation of smoking cessation interventions in the workplace.

Improving the health status of older workers through encouraging smoking cessation (Sachs-Ericsson et al. 2009), could improve their work-related outcomes and sustained employability, thereby increasing the probability they will be able to work up till retirement age (Nicita-Mauro et al. 2008). Several studies have found that smoking cessation reduces the risk of all-cause mortality (Doll et al. 1994; Kenfield et al. 2008). For those who quit smoking early in their adult life, mortality decreased to the level of a never smoker (Doll et al. 1994; Kenfield et al. 2008). Smokers who quit after the age of 45 year experienced an increase in survival compared to sustained smokers (Doll et al. 1994). In several studies attempting to determine the effect of smoking status, a distinction is made between non-smokers, current smokers, and former smokers (Bunn et al. 2006; Suwa et al. 2017; Wacker et al. 2013). However, since former smokers could have been abstinent for several decades, this category does not necessarily reflect the effects of smoking cessation.

Few studies have looked into the effect of smoking cessation on work-related outcomes. One study found that within 0–4 years after smoking cessation, absence and productivity loss decreased (Baker et al. 2017). According to another study, after cessation, objective measures of productivity decreased in the first years, but exceeded the productivity of sustained smokers 1–4 years after cessation (Halpern et al. 2001). Therefore, more research on the association between smoking cessation and work-related outcomes is needed.

Therefore, the aims of this study are to assess (1) the effect of sustained smoking, and (2) the effect of quitting on sickness absence, productivity loss and work ability among older workers. We hypothesize that comparing sustained smoking to non-smoking, sustained smoking is associated with higher levels of sickness absence and productivity loss, and a lower level of work ability, and that comparing quitting to sustained smoking, quitting is associated with lower levels of sickness absence and productivity loss, and a higher level of work ability.

## Methods

### Study design and participants

**Fig. 1:**
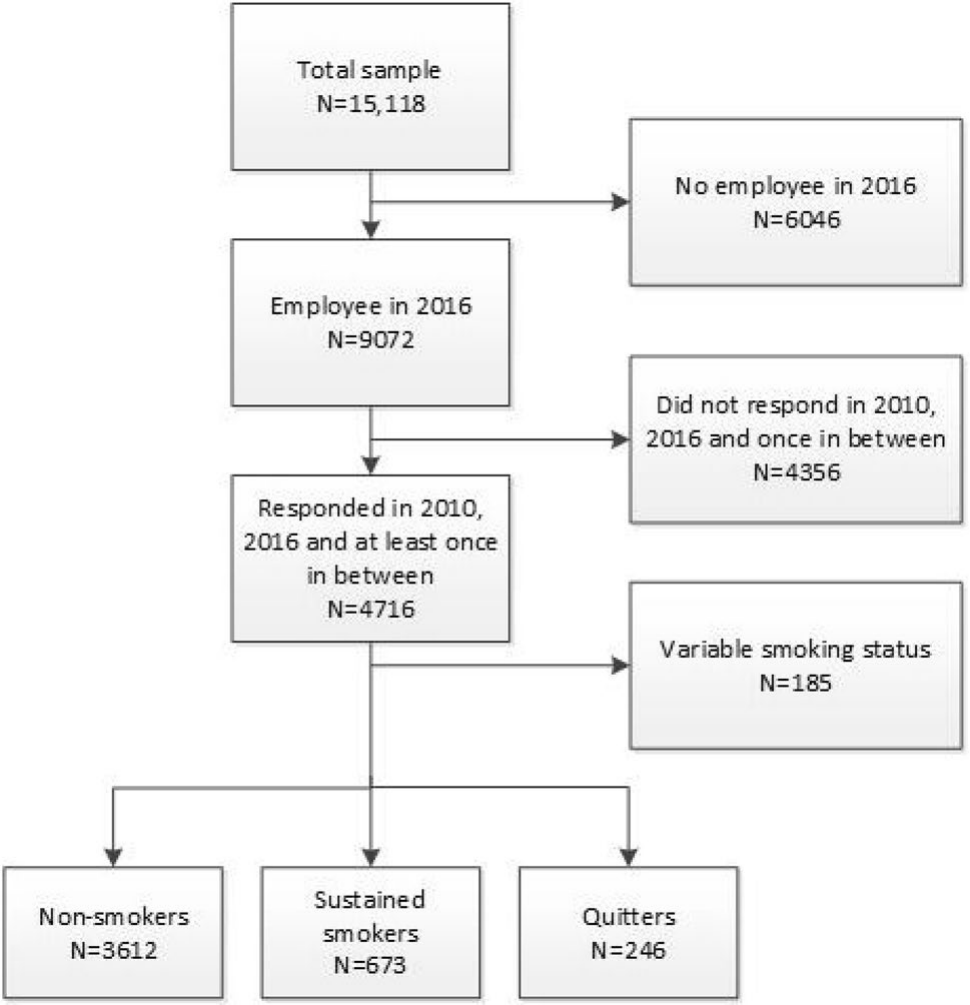
Flow chart of participant inclusion

This study used data from the Study on Transitions in Employment, Ability and Motivation in the Netherlands (STREAM) (Ybema et al. 2014). This is a prospective cohort study consisting of a total sample of 15,118 Dutch persons aged 45–64 years (Ybema et al. 2014) (Fig. 1). The STREAM cohort was set up to provide insight in factors that influence working until retirement in a healthy and productive manner (Ybema et al. 2014). STREAM participants completed online questionnaires in 2010, 2011, 2012, 2013, 2015 and 2016 on topics related to employment, work characteristics, health status, sickness absence, productivity loss, and work ability. For the present study, we excluded STREAM participants when they were not employed in 2016 (T6) (*N* = 6046), when they did not participate in the questionnaire in 2010 (T1), 2016 (T6), and at least once in between (2011 2012, 2013, 2015 (T2-T5) (*N* = 4356), and when their smoking status could not be categorized into non-smoker, sustained smoker or quitter, due to frequent changes in their smoking status (*N* = 185). The analytic sample consisted of 4531 individuals (Fig. 1).

Comparisons between respondents who participated in all questionnaires versus those who missed one or more follow-up questionnaire showed some selective loss to follow-up. Due to the large sample size, differences were statistically significant but very small. For all variables, Cohen’s *d* was smaller than 0.201, except for age (0.218), indicating that selective follow-up will not seriously have biased the results (van den Heuvel et al. 2016).

## Measures

### Outcomes

Individual sickness absence percentage was calculated by dividing the self-reported days on sick leave in the past 12 months by the number of potential working day (i.e., corrected for part-time work and with full-time work defined as 215 working days per year). This represents the proportion of worktime missed due to illness in the past year. We also dichotomized sickness absence percentage into any sickness absence (“yes” or “no”) to make our findings comparable with recent systematic reviews on smoking and sickness absence.

Productivity loss at work was assessed in all surveys using a question on the self-assessed quantity of executed work in the last 4 weeks compared to usual (Ybema et al. 2014). Answers were categorized in 1 = ”less compared to normal”, 2 = ”similar to normal”, and 3 = ”more compared to normal”. We dichotomized the answers into 0 = ”no productivity loss, i.e., similar or higher productivity compared to normal” and 1 = ”productivity loss, i.e., lower productivity compared to normal”.

Work ability in relation to job demands was assessed at all survey waves using the second dimension of the work ability index (WAI 2) (Ilmarinen 2007, 2009; Tuomi et al. 2007). The Work Ability Index (WAI), used to assess work ability, has been widely researched and is considered to have high predictive validity and cross-national stability (Lundin et al. 2017; Radkiewicz and Widerszal-Bazyl 2005). The WAI 2 consists of two questions that subjectively assess current work ability in relation to the (a) physical and (b) mental demands of their job on a scale from 1 = ”very poor” to 5 = ”very good”. The scores were combined into dimension 2 of the work ability index, work ability in relation to job demands, ranging from 2 = ”very bad” to 10 = ”excellent” (van den Heuvel et al. 2016). Previous research has shown that the WAI 2 is strongly associated with both the overall WAI and other constructs that are related to work ability (Ebener et al. 2019). In contrast to the outcome variables sickness absence percentage and productivity loss, where increases can be regarded as ‘negative’, an increase in work ability can be regarded as ‘positive’.

### Smoking status

Smoking status was assessed at all survey waves by the question whether participants were a current smoker, former smoker, or non-smoker. Participants were divided in three categories based on their smoking status. The first category consisted of non-smokers, those who reported not to smoke at all completed surveys. The second category consisted of sustained smokers. These smokers reported to smoke during at least 75% of all completed surveys (meaning that depending on their participation rate, they could report being a former smoker once). The third category, ‘quitters’ consisted of people who reported being a current smoker at T1 and reported being a former smoker in their last two completed surveys.

### Potential confounders

We included the following baseline variables as potential confounders: age, gender, educational level, physical health, mental health, BMI, and job demands. Age was measured on a continuous scale and gender was measured dichotomously. Educational level was divided in three groups (low, middle, high), based on the highest reported education type. Physical health was measured using the SF12 physical composite scale score (Ware et al. 1996). Mental health was measured using the SF12 mental composite scale score (Ware et al. 1996). Body Mass Index (BMI) was determined through self-reported body weight and height of participants and dichotomized into ≤ 25 and > 25 to distinguish between under/normal bodyweight and overweight participants. Job demands was measured using four questions on whether the participant had to work very fast, extra hard, do a lot of work, or had to do hectic work (four items, Cronbach’s *α* = 0.87) (van den Heuvel et al. 2016).

### Statistical analysis

First, all variables were analyzed descriptively to report baseline characteristics of non-smokers, sustained smokers and quitters. Afterwards, the associations between smoking status (based on T1–T6) and sickness absence, productivity loss, and work ability (as reported at T6) were analyzed using logistic (for the outcome regarding productivity loss) and linear (for the outcomes regarding sickness absence and work ability) regression analyses. Even though work ability was an ordinal outcome measure, since in total 17 different scores were reported (from 2 to 10 with steps of 0.5), we used linear regression analyses. Six different regression analyses were performed, two for each of the three outcomes, one assessing the effects of sustained smoking with non-smoking as reference group (aim 1) and one assessing the effects of quitting with sustained smoking as reference group (aim 2). Furthermore, since we expected physical health status to be of influence on the decision to quit smoking, we tested for interactions between smoking cessation and physical health at baseline. Comparing quitting versus sustained smoking, we found significant interactions between physical health and the outcomes. Therefore, we stratified our analysis based on the median physical health score (54.88) of the study population. For each analysis, we built four models: (0) univariate analyses, (1) with demographic variables (age, gender and educational level), (2) with demographic variables and job demands and (3) with demographic variables, job demands and health status (physical health, mental health and BMI). Since health status could be both a confounder and a mediator in the relation between smoking status and work-related outcomes, we added these variables to the final model. The analyses were performed using SPSS statistical software version 22.

## Results

### Sustained smoking versus non-smoking

Baseline characteristics for sustained smokers (*N* = 673) and non-smokers (*N* = 3612) are shown in Table 1. Table 2 shows the longitudinal effects of sustained smoking on sickness absence percentage, productivity loss, and work ability. Comparing sustained smokers to non-smokers, we found that sustained smokers had a somewhat higher (but not statistically significant) sickness absence percentage. After addition of health status in model 3, the association with sickness absence changed substantially compared to model 2 (OR 0.82, 95% CI − 0.35–2 .00, *p* 0.17 vs. 1.01, 95% CI – 0.16–2.17, *p* 0.09). We did not find statistically significant differences in productivity loss (OR 0.82, 95% CI 0.60 – 1.13, *p* 0.23) and work ability (0.05, 95% CI −. 05–0.15, *p* 0.32) comparing sustained smokers to non-smokers.

**Table 1:** Characteristics of non-smoking, sustained smoking and quitting participants at baseline

	Non-smokers (*N* = 3612)			Sustained smokers (*N* = 673)			Quitters (*N* = 246)		
	Mean	SD	%	Mean	SD	%	Mean	SD	%
Age	52.21	4.64		52.09	4.45		52.24	4.41	
Male			56.9			57.4			53.3
Low education			23.4			35.2			29.3
Physical health	52.31	7.25		51.79	7.05		51.71	6.92	
Mental health	52.32	8.09		51.99	8.09		51.95	8.31	
BMI > 25			65.8			54.4			59.8
Job demands	3.15	0.74		3.25	0.73		3.24	0.75	
Sickness absence %	4.02	12.26		4.53	12.25		4.12	12.23	
Productivity loss			7.2			7.8			3.0
Work ability	8.26	1.13		8.24	1.11		8.20	1.11	

*N* number; *SD* standard deviation; *BMI* body mass index

**Table 2:** Longitudinal effects of sustained smoking on sickness absence, productivity loss, and work ability with non-smokers as reference group, *N* = 4285

	Univariate	Model 1 (+ demographics)	Model 2 (+ demographics, job demands)	Model 3 (+ demographics, job demands and health status)
	Coef/OR [95% CI]	Coef/OR [95% CI]	Coef/OR [95% CI]	Coef/OR [95% CI]
Sickness absence % (Coef)^1^	0.88 [− 0.28–2.03]	0.80 [− 0.36–1.96]	0.82 [− 0.35–2.00]	1.01 [− 0.16–2.17]
Productivity loss (OR)^2^	0.81 [0.60–1.10]	0.83 [0.61–1.12]	0.85 [0.62–1.15]	0.82 [0.60–1.13]
Work ability (Coef)^1^	0.01 [− 0.09–0.11]	0.02 [– 0.08–0.12]	0.04 [− 0.06–0.14]	0.05 [− 0.05–0.15]

*OR* odds ratio; *Coef* coefficient; *CI* confidence interval

^1^Results based on linear regression modelling

^2^Results based on logistic regression modelling

### Quitting versus sustained smoking

Baseline characteristics for quitters (*N* = 246) and sustained smokers (*N* = 673) are shown in Table 1. Table 3 shows the longitudinal effects of smoking cessation on sickness absence, productivity loss and work ability, stratified for physical health score. For employees with a relatively high physical health score at baseline, comparing quitters to sustained smokers, we found substantially, but not statistically significant, higher odds of productivity loss for quitters (OR 2.23, 95% CI 0.94−5.31, *p* 0.07). We did not find statistically significant differences in sickness absence percentage (0.10, 95% CI − 2.67–2.87, *p* 0.94), and work ability score (− 0.10, 95% CI − 0.36−0.16, *p* 0.43) (Table 3).

**Table 3:** Longitudinal effects of smoking cessation on sickness absence, productivity loss, and work ability, stratified for physical health with sustained smokers as reference group, *N* = 919

	Univariate	Model 1 (+ demographics)	Model 2 (+ demographics, job demands)	Model 3 (+ demographics, job demands, health status)
	Coef/OR [95% CI]	Coef/OR [95% CI]	Coef/OR [95% CI]	Coef/OR [95% CI]
Employees with relatively high physical health score at baseline				
Sickness absence % (Coef)^1^	0.06 [− 2.55–2.66]	0.24 [− 2.392–0.87]	0.33 [− 2.42–3.08]	0.10 [− 2.67–2.87]
Productivity loss (OR)^2^	2.04 [0.88–4.72]	2.05 [0.88–4.79]	2.24 [0.94–5.32]	2.23 [0.94–5.31]
Work ability score (Coef)^1^	− 0.09 [− 0.34–0.16]	− 0.10 [− 0.35–0.16]	− 0.10 [− 0.36–0.16]	− 0.10 [− 0.36–0.16]
Employees with relatively low physical health score at baseline				
Sickness absence % (Coef)^1^	2.78 [− 0.91–6.47]	2.82 [− 0.87–6.52]	2.98 [− 0.83–6.79]	2.53 [− 1.29–6.34]
Productivity loss (OR)^2^	1.40 [0.77–2.56]	1.43 [0.78–2.61]	1.30 [0.70–2.41]	1.26 [0.66–2.39]
Work ability score (Coef)^1^	− 0.30* [− 0.57–0.03]	− 0.30* [− 0.57–0.03]	− 0.30* [− 0.58–0.02]	− 0.31* [− 0.57–0.05]

*OR* odds ratio; *Coef* coefficient; *CI* confidence interval

^1^Results based on linear regression modelling

^2^Results based on logistic regression modelling

For employees with a relatively low physical health score at baseline, comparing quitters to sustained smokers, we found a statistically significant lower work ability score for quitters (− 0.31, 95% CI − 0.57–0.05, *p* 0.02). We did not find statistically significant differences in sickness absence percentage (2.53, 95% CI − 1.29–6.34, *p* 0.19) and productivity loss (OR 1.26, 95% CI 0.66–2.39, *p* 0.48).

## Discussion

### Summary of main findings

In contrast to our hypotheses, we found no differences in sickness absence percentage, productivity loss and work ability score for sustained smokers compared to non-smokers, although sickness absence percentage was somewhat higher (but not statistically significant) for sustained smokers. Comparing quitters to sustained smokers, we found less favorable results for quitters in two out of the six associations. Among individuals with a relatively poor physical health at baseline, work ability was significantly lower for quitters. However, we found no significant differences in sickness absence and productivity loss.

### Methodological considerations

This study has a number of strengths. First, few studies have determined the effect of both sustained smoking and smoking cessation on work-related outcomes (Baker et al. 2017; Halpern et al. 2001). This study is among the first to determine the effects of recent smoking cessation (Baker et al. 2017), which is more relevant from the perspective of an employer considering to implement a smoking cessation intervention in the workplace. Furthermore, we are the first to provide insight in the relation between smoking cessation and work-related outcomes in a population of older employees.

A potential limitation of this study is the measurement of smoking status. Evidence on the sensitivity and specificity of self-reported smoking status is mixed. A meta-analysis comparing self-reported smoking status with biochemical validation found a generally high sensitivity (mean 87.5%) and specificity (mean 89.2%) of self-reported smoking status (Patrick et al. 1994). However, a more recent systematic review found an underestimation of smoking prevalence and varying sensitivity levels (Gorber et al. 2009). For our study, this means that we could have misclassified sustained smokers as quitters and vice versa, which might have reduced sensitivity in our sample, thereby underestimating the effects of sustained smoking and smoking cessation on work-related outcomes.

The instruments used to measure work-related outcomes were also based on self-reporting. According to literature, agreement between self-reported and recorded sickness absence days per year is relatively good (Ferrie et al. 2005). For productivity loss, few studies are available that compared self-reported with objective measures. One study found large individual differences in self-reported and objective daily work productivity reports (Finkelstein et al. 2006). Furthermore, accuracy and representativeness of self-reported work productivity depends on the recall period (Brooks et al. 2010). In the present study the recall period was 4 weeks, which is probably is good balance between accuracy and representativeness. Finally, since work productivity is influenced by multiple factors, smoking-associated work productivity might not be fully captured by our outcome variables.

### Interpretation of results

In contrast to our hypothesis, we found no statistically significant differences in work-related outcomes for sustained smokers compared to non-smokers. This suggests that among older employees, sustained smokers do not have more negative work-related outcomes compared to non-smokers. These results correspond with one study on the relation between smoking status and work ability (Tuomi et al. 2001), but are in contrast to several other studies on this relation (Kaleta et al. 2006; Tuomi et al. 1991). Furthermore, our findings are in contrast to several studies on the association between smoking status and work productivity (Berman et al. 2014; Bunn et al. 2006; Halpern et al. 2001; Sherman and Lynch 2013).

We found a higher sickness absence percentage for sustained smokers compared to non-smokers. However, this difference was not statistically significant. This is in contrast to two systematic reviews on the relation between sustained smoking and sickness absence (Troelstra et al. 2020; Weng et al. 2013), but in correspondence with several individual studies included in the systematic review which also did not find statistically significant associations between smoking and sickness absence (Boles et al. 2004; Karlsson et al. 2010; Kivimäki et al. 1997; Pai et al. 2009). A potential explanation for our results could be over-adjustment for health status. After controlling for health at baseline in model 3, the association with sickness absence changed substantially compared to model 2 but remained not statistically significant. As an additional analysis we dichotomized sickness absence and found an odds ratio of 1.10 (95% CI 0.92–1.30) comparing sustained smoking with non-smoking. This 95% confidence interval, when interpreted as a relative risk, is lower but overlaps with the confidence intervals of risk of sickness absence for sustained smokers compared to non-smokers as reported in two recent systematic reviews (1.24–1.39 and 1.25–1.41, respectively) (Troelstra et al. 2020; Weng et al. 2013). This indicates that even though our findings are unexpected, they are within the range found by other studies.

Comparing quitters to sustained smokers, we found less favorable results for quitters in two of six associations. For work ability and quitting among individuals with a relatively poor physical health, this association was statistically significant. Several studies suggest that even though smoking cessation will increase health status and reduce mortality within a few years after cessation, it might take much longer until they are up to the level of a never smoker (Doll et al. 1994; Kenfield et al. 2008). One study found that among those who had quit smoking for less than 1 year, productivity was lower compared to sustained smokers, whereas their productivity increased and exceeded the productivity of current smokers 1–5 years after quitting smoking (Halpern et al. 2001). This study suggested that there may be a “dose–response” relationship between work productivity and years of cessation. Another study found that within 5 years of having quit smoking, work impairment was lower compared to sustained smokers; however, this study did not distinguish between the first and later years of follow-up (Baker et al. 2017). Therefore, a possible explanation for our unexpected results might be that the relatively short time our participants had quit smoking (1–5 years) is too short for the beneficial effects of smoking cessation to be manifested in this sample.

Another explanation could be that participants who were concerned about their deteriorating health status were more likely to attempt to quit smoking (Freund et al. 1992; Sachs-Ericsson et al. 2009). Therefore, while both sustained smokers and quitters might experience a relatively poor physical health compared to non-smokers, quitters might be more likely to experience their health as having an impact on their work ability. Furthermore, quitters might have decided to quit smoking due to actual health problems, which they developed during the 6 years between T1 and T6. It has been suggested that people that are trying to quit smoking might have more unfavorable work-related outcomes compared to smokers that are not trying to quit (Baker et al. 2017). We stratified our sample based on the median physical health at the first measurement. However, changes in health status that occurred in the following years might still be of influence and could have encouraged a selective part of our study sample to quit smoking and might explain lower work-related outcomes in quitters compared to sustained smokers.

In this study we were unable to find support for a positive effect of implementing smoking cessation interventions among older employees in terms of work-related outcomes. However, the general body of evidence on the benefits of smoking cessation leaves no doubt towards its importance from a public health perspective and on an individual level (Jha et al. 2013; Parrott and Godfrey 2004). Employers have the opportunity to play an influential role in improving their employees’ health, since they can identify, access and support their smoking employees with relative ease (Carroll et al. 2013; Fishwick et al. 2013).

## Conclusions

Sustained smokers did not have higher sickness absence, productivity loss, and lower work ability compared to non-smokers. Quitters did not have lower sickness absence and productivity loss compared to sustained smokers, but quitters might have a lower work ability compared to sustained smokers. The benefits of smoking cessation for employers might a take a longer time to develop.
